# Whole‐exome sequencing identified mutational profile of a case with T‐cell chronic lymphocytic leukemia

**DOI:** 10.1002/ccr3.3149

**Published:** 2020-07-30

**Authors:** Kenji Nozaki, Takafumi Yokota, Eri Itotagawa, Kazuhito Tsutsumi, Shinsuke Kusakabe, Yoichiro Morikawa, Jiro Fujita, Kentaro Fukushima, Tetsuo Maeda, Hirohiko Shibayama, Naoki Hosen, Atsushi Kumanogo, Yuzuru Kanakura

**Affiliations:** ^1^ Department of Hematology and Oncology Osaka University Graduate School of Medicine Suita Japan; ^2^ Department of Respiratory Medicine and Clinical Immunology Graduate School of Medicine Osaka University Suita Japan

**Keywords:** haematology, T‐cell chronic lymphocytic leukemia

## Abstract

We believe that our report and further case reports on T‐cell chronic lymphocytic leukemia with genetic profile will contribute to the molecular classification of this rare but distinct disease.

## Graphical abstract

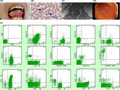

## 1 INTRODUCTION

Morphological evaluations are insufficient for T‐cell form of chronic lymphocytic leukemia (T‐CLL) diagnosis. Here, we detected 13 mutations by whole‐exome sequencing and confirmed them by Sanger sequencing. This report reveals the genetic profile of a T‐CLL patient and contributes to the molecular classification of this rare but distinct disease.

Since the term T‐cell chronic lymphocytic leukemia (T‐CLL) was first used in 1975,^1^ extensive case reports without common genetic or immunophenotypic profiles have prompted debate regarding its nature and existence. As a result, T‐CLL is now described as a small‐cell variant of T‐cell prolymphocytic leukemia (T‐PLL‐sv) or mature T‐cell leukemia (MTCL), unclassified.^2^ However, the classification of indolent MTCL remains controversial because morphological evaluations alone are insufficient to predict clinical outcomes. In this report, we characterized a case of T‐CLL by whole‐exome sequencing (WES) to contribute to the molecular classification of this disease.

## 2 PATIENTS AND METHODS

**FIGURE 1:**
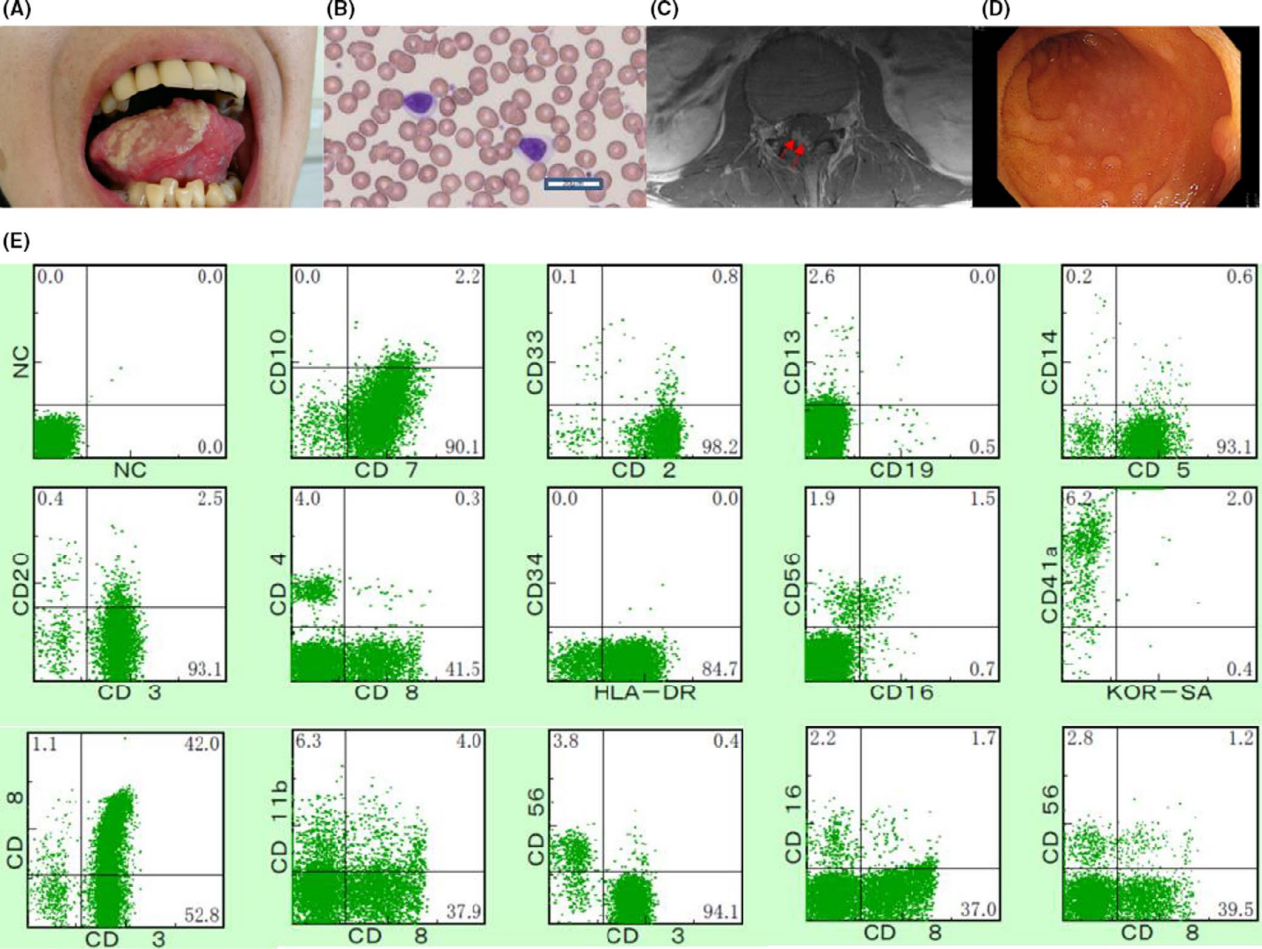
A, Refractory oral ulcers. B, Peripheral blood smear of leukemic cells. C, Flow cytometric analysis of the bone marrow. D, MRI with contrast of the lumbar. (arrow: enhanced lesion in the L4‐S1 nerve root). E, Colon ulcers

A 33‐year‐old man with refractory oral ulcers was diagnosed with T‐CLL based on CD8‐positive T lymphocytosis and the TCR cβ, Jγ rearrangement 18 years ago. After 7 years of careful monitoring, he developed uveitis and multiple colon ulcers. Treatment with immunosuppressive agents was initiated for the possible diagnosis of Behçet's disease. Cyclosporine induced the remission of lymphocytosis for 5 years. However, the treatment was temporarily stopped 3 years ago when the patient presented with severe headaches and an intractable tongue ulcer (Figure 1A), since cyclosporine generally worsens neurological symptoms of Behçet's disease.^3^

A hematological examination revealed a white blood cell (WBC) count of 10 080/µL with 7.0% mature lymphocytes (CD4: 5.8%, CD8: 43.3%), which were small‐to‐middle sized with a high nuclear/cytoplasmic ratio, lacking a nucleolus and cytoplasmic granules (Figure 1B). A serological test for human T‐lymphotropic virus type‐1 was negative. A serological analysis of Epstein‐Barr virus revealed a past infection. Magnetic resonance imaging (MRI) of the head and lumbar region without contrast revealed no abnormalities. Cerebrospinal fluid (CSF) showed elevated cell counts (53/μL) with 100% lymphocytes.

After admission for further examination, the patient became febrile and developed symptoms of restless leg syndrome in the right leg, including paresthesia worsening at night. The lymphocyte count increased gradually (highest WBC count, 33 370/µL, with 80.5% mature lymphocytes). Southern blotting of peripheral blood (PB) and bone marrow (BM) showed a TCR cβ1 and Jγ rearrangement. CSF showed remarkably elevated cell counts (560/μL), characterized by small‐to‐moderate atypical lymphocytes. BM examination revealed the proliferation of small lymphocytes classified as CD3+ CD4− CD8+ dominant and negative for TIA‐1 and granzyme B, without chromosome abnormalities. Flow cytometry of BM showed that cells were CD3+ CD4− CD5+ CD7+ CD8+ CD16− CD19− CD56− (Figure 1C). MRI with contrast of the lumbar revealed an enhanced lesion in the L4‐S1 nerve root (Figure 1D), compatible with the leg symptoms. The patient underwent a total colonoscopy due to abdominal pain and biopsied specimens of colon ulcers indicated the invasion of a T‐cell neoplasm (Figure 1E). The patient was diagnosed with indolent MTCL, unclassified with central nerve system (CNS) and gastrointestinal (GI) invasion.

Cyclosporine was resumed, along with intrathecal chemotherapy with methotrexate, cytarabine, and dexamethasone. Lymphocytosis improved, and leg symptoms were relieved. The disease was maintained in an indolent stage under cyclosporine administration with minimal neurological symptoms.

We used WES to identify somatic mutations in leukemic cells. A commercial platform (Agilent) was used to prepare libraries for genomic DNA from the leukemic (CD8+) and nonleukemic (CD8−) cells obtained from BM samples. Sequencing was performed using an Illumina HiSeq 2500 sequencer. We performed variant calling with GATK, annotation with ANNOVAR, and prioritization to identify somatic and functional mutations.

## 3 RESULTS AND DISCUSSION

We identified 13 mutations, including nonsynonymous single nucleotide variant (SNVs) in *ASXL1*,*CD247*,*DDX3X*,*GNAZ*,*HSPA12A*,*LRIF1*,*SLCO1B7*,*TMEM121*,*TYK2*, and *VWA5A*, frameshift deletions in *LINC00403* and *ZNF772*, and a nonframeshift insertion in *CLCN7* (Table 1), without genetic characteristics for LGL (*STAT3* or *STAT5B*) or T‐PLL (*TCL‐1* or *MTCP1* or *ATM*). We confirmed all variants, other than deletions by Sanger sequencing (Figure S1).

**TABLE 1:** List of the 13 mutations found in the leukemic cells

Gene	Chromosome	Start	End	Reference	Alteration	Mutation type
ASXL1	20	31 023 427	31 023 427	A	G	Nonsynonymous SNV
CD247	1	167 409 938	167 409 938	T	C	Nonsynonymous SNV
CLCN7	16	1 510 453	1 510 453	‐	CGAAGGCGG	Nonframeshift insertion
DDX3X	X	41 202 485	41 202 485	T	G	Nonsynonymous SNV
GNAZ	22	23 465 397	23 465 397	C	T	Nonsynonymous SNV
HSPA12A	10	118 590 147	118 590 147	G	A	Nonsynonymous SNV
LINC00403	13	112 763 198	112 763 199	CA	‐	Frameshift deletion
LRIF1	1	111 495 034	111 495 034	T	C	Nonsynonymous SNV
SLCO1B7	12	21 229 431	21 229 431	C	T	Nonsynonymous SNV
TMEM121	14	105 995 176	105 995 176	T	G	Nonsynonymous SNV
TYK2	19	10 476 512	10 476 512	C	T	Nonsynonymous SNV
VWA5A	11	124 007 723	124 007 723	C	A	Nonsynonymous SNV
ZNF772	19	57 988 654	57 988 666	GCCCATCGGCTCA	‐	Frameshift deletion

Abbreviation: SNV, single nucleotide variant.

Strikingly, we found a nonsynonymous SNV in *CD247*. This gene encodes T‐cell receptor zeta, which forms the T‐cell receptor (TCR)–CD3 complex. In addition to contributing to T‐cell activation, CD3 zeta participates in intrathymic T‐cell differentiation.^4^ As CD3 zeta plays a key role in receptor expression and signal transduction,^5^ it is possible that the *CD247* mutation contributes to the pathogenesis of the disease by altering the stimulation of TCR, which triggers T‐cell activation. It is plausible that cyclosporine effectively inhibited T cells activated via TCR signaling.^6^ Nonsynonymous SNVs in *DDX3X* and *TYK2* may also be important because their dysregulation has been implicated in tumorigenesis.^7^

We propose that the classification of indolent MTCL must not be based purely on morphological criteria, but should be substantiated by underlying molecular/genetic features as well as clinical manifestations. We describe a unique clinical presentation, detailed immunohistological features, and, most importantly, WES data for this rare disease.

The 18‐year clinical course was characterized by the following key findings. (a) The leukemia was managed by careful monitoring for 7 years from the initial diagnosis. (b) Cyclosporine controlled the disease for longer than 7 years, suggesting that the disease behaves more like large granular lymphocyte (LGL) leukemia than like T‐cell prolymphocytic leukemia (T‐PLL). However, the morphology of small‐to‐middle sized mature lymphocytes without azure granules or a nucleolus differed from those of LGL and T‐PLL. (c) Without cyclosporine, the disease behaved aggressively, causing lymphocytosis with fever and CNS and GI invasion. (d) CNS involvement caused symptoms of restless leg syndrome with favorable neurological prognosis. CNS involvement of indolent MTCL is extremely rare and its presentation of involvement is quite unusual. The moderate neurological damage was suggestive of an inflammatory reaction of the involved sites, considering that the leukemia was accompanied by some inflammatory symptoms, such as oral ulcers, uveitis, and colon ulcers, of which only colon ulcers were pathologically diagnosed as T‐cell neoplasm invasion.

This T‐CLL case can hardly be considered as a variant of T‐PLL and can only be described as indolent MTCL, unclassified as of now. Newly identified mutations extended our knowledge of the molecular basis and provide insight into the pathogenesis of this disease. We believe that our report and further case reports revealing the genetic profile will contribute to the molecular classification of this rare but distinct disease.

## CONFLICT OF INTEREST

None declared.

## AUTHOR CONTRIBUTION

KN: performed the research. KN and TY: analyzed the data and wrote the paper. All authors: approved the final version of the manuscript.

## Supporting information

Fig S1

Fig S1‐cap
