# Supplementary figures and images for: Whole‐exome sequencing identified mutational profile of a case with T‐cell chronic lymphocytic leukemia
Source: Clin Case Rep. 2020 Jul 30;8(11):2251–4. doi: 10.1002/ccr3.3149 (PMC7669389; doi:10.1002/ccr3.3149)

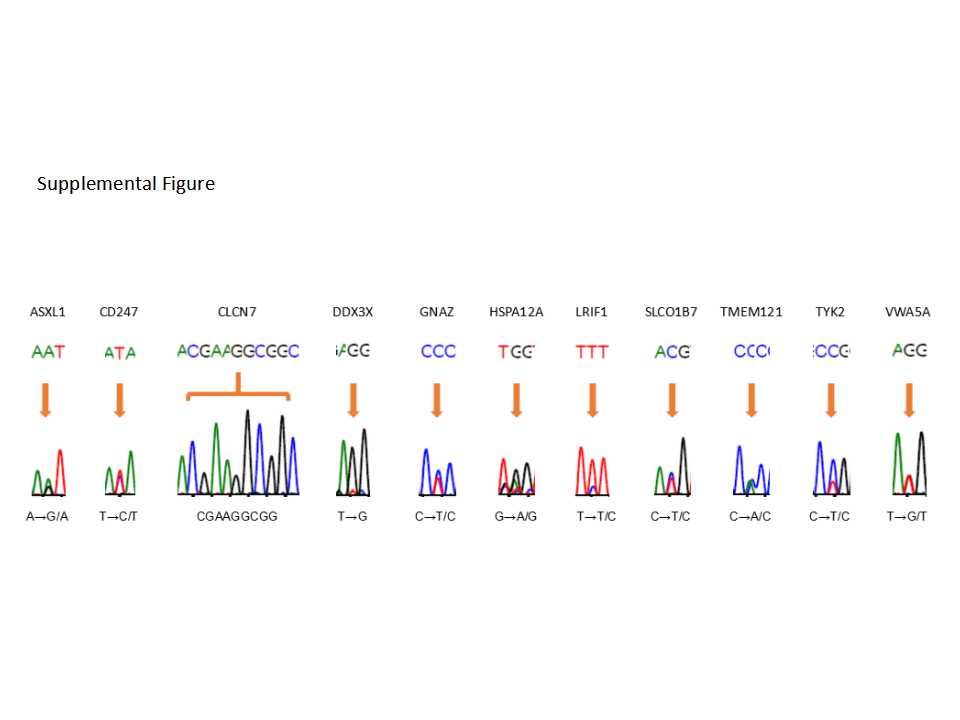

Supplement: Supplementary file 1 — Fig S1 [file CCR3-8-2251-s001.jpg]
