# Supplementary material for: Whole‐exome sequencing identified mutational profile of a case with T‐cell chronic lymphocytic leukemia
Source: Clin Case Rep. 2020 Jul 30;8(11):2251–4. doi: 10.1002/ccr3.3149 (PMC7669389; doi:10.1002/ccr3.3149)
Supplement: Supplementary file 2 — Fig S1‐cap [file CCR3-8-2251-s002.docx]

**Supplemental Figure S1: Sanger sequencing chromatograms.** Chromatograms of TMEM121 and VWA5A are shown in reverse reads.
